# A neuroimmune framework for understanding adolescent stress and risk of alcohol misuse

**DOI:** 10.1016/j.bbih.2026.101242

**Published:** 2026-04-17

**Authors:** Léa Aeschlimann, Narimane Bouzourène, Clara Rossetti, Benjamin Boutrel

**Affiliations:** Center for Psychiatric Neuroscience, Department of Psychiatry, Lausanne University Hospital and University of Lausanne, Switzerland

**Keywords:** Adolescence, Alcohol use disorder, Addiction vulnerability, Neuroinflammation, Stress, Microglia, HPA axis, Gut-microbiota-brain axis

## Abstract

Alcohol use disorder (AUD) represents a critical public health challenge, with early-life adversity conferring heightened risk for compulsive drinking patterns. Alcohol has long been known to exert direct effects on developing neurotransmitter systems, targeting among others GABAergic, glutamatergic, and dopaminergic signaling. Recent evidence however indicates that early adversity also primes microglial activation and establishes chronic low-grade neuroinflammation, disrupting maturation of prefrontal-limbic-striatal circuits governing executive control and affect regulation. Hence, alcohol use may emerge in adolescents as a maladaptive coping mechanism, transiently alleviating stress-related dysphoria while exacerbating neuroimmune and neurocircuit dysfunction. While traditional neurocentric models convincingly depict how repeated withdrawal episodes may unmask this underlying vulnerability, precipitating relapse cycles that consolidate compulsive use, they inadequately explain the persistence and treatment resistance characteristic of adolescent-onset alcohol misuse because they fail to account for the peripheral biological systems that amplify central vulnerability. The gut-microbiota-brain axis represents one such amplifier: stress- and alcohol-related perturbations in barrier integrity and immunometabolic signaling increase peripheral inflammatory load reaching the brain, intensifying neuroimmune tuning of still-maturing control circuits. Integrating these central and peripheral processes reframes adolescent alcohol vulnerability as a systems-level phenomenon embedded within developmental and inflammatory biology, rather than a disorder of reward circuitry alone. This developmental framework suggests that adjunctive therapeutic strategies combining targeted neuroimmune modulation, behavioral intervention, and ecological stabilization during critical developmental windows may offer superior outcomes over conventional reward-focused pharmacotherapies. Realizing this potential will require biomarker-driven risk stratification, precision medicine approaches, and careful developmental consideration of intervention timing**.**

## 1 Introduction

Compulsive alcohol consumption in at-risk adolescents does not signify a deficit of willpower, but rather the behavioral endpoint of stress-induced neurobiological recalibration involving inflammatory signaling and affective regulatory circuitry. Within this framework, alcohol use may emerge as a compensatory regulatory behavior attempting to stabilize dysregulated emotional and inhibitory systems. Recent decades have transformed the conceptualization of addiction by shifting from brain-centric models to a systems biology approach, integrating peripheral physiology and computational analytics. Understanding this shift requires integrating historical milestones with advances in neuroimmunology, gut–brain signaling, and machine-learning approaches to vulnerability and stratification. Initial addiction research emphasized specific brain regions and neurotransmitter systems, hence framing addiction as primarily rooted in neural circuits. This focus, dominant throughout the mid-20th century, provided critical neurobiological insights but limited the understanding of addiction's complex and multifactorial nature. Recent advances in systems biology and neurotechnology highlight dynamic interactions between the central nervous system (CNS) and peripheral systems, including metabolic, immune, and endocrine pathways, with particular attention to the gut-brain axis.

The 1960s-1970s marked the rise of organ-centric addiction models, catalyzed by molecular discoveries: opioid receptors (Pert and Snyder, 1973), endogenous opioids (Hughes et al., 1975) (building on conceptual work of Goldstein et al., 1971) and dopamine receptors (Seeman and Lee, 1975; Burt et al., 1975), solidifying addiction's neurochemical underpinnings. These studies informed early pharmacological interventions (Dole and Nyswander, 1965; Dole et al., 1966) and reinforced the dopamine hypothesis, framing addiction as the hijacking of endogenous reward circuitry and guiding brain-centric therapeutic development. Recognizing limitations of neural-centric views, research from the 1970-1980s onward embraced integrated models. Theories of anhedonia (Wise, 1982) and neurotransmitter system dysregulation (Iversen, 1971) revealed addiction's chronic and relapsing nature as emerging from distributed circuit dysfunction. Subsequent conceptual advances, including negative reinforcement models (Markou and Koob, 1991) and the “hedonic dysregulation and allostasis” theory (Koob and Moal, 1997), reframed addiction as a disorder of systemic imbalance, implicating reward and stress circuits alongside HPA-axis dysregulation. Parallel work on cortico-striatal learning mechanisms further demonstrated the progression from voluntary drug use to compulsive behavioral patterns (Everitt and Robbins, 2005). These insights laid the framework for viewing addiction as involving complex crosstalk between multiple neural and peripheral systems. In the 21st century, neuroimaging Volkow et al. (1996) and molecular biology Nestler (2001) provided new dimensions, linking circuit-level adaptations to enduring changes in transcriptional and stress-related regulatory pathways. McEwen's research McEwen (2000) on stress-induced plasticity further anchored addiction models in systemic adaptation, highlighting the relevance of peripheral endocrine and immune signaling. Clinical translation by O'Brien and McLellan (1996) (pharmacological interventions like naltrexone) bridged mechanistic neuroscience with evidence-based treatment approaches. These converging streams highlight that neither molecular, circuit-level, nor behavioral models alone can explain addiction persistence, only multi-level integration suffices. Extending these integrative models beyond central circuitry, increasing attention has focused on peripheral systems capable of shaping neurobehavioral trajectories. The gut-brain axis represents a key interface linking environmental exposures, immune signaling, and central nervous system function. Foundational experimental studies demonstrated that gut microbiota influence brain development, immune signaling, and behavior (Hsiao et al., 2013; Diaz et al., 2011), supporting bidirectional microbiota-brain communication (Mayer, 2011; Cryan and Dinan, 2012 for integrative overviews). These interactions are increasingly implicated in adolescent vulnerability to addiction-relevant phenotypes, positioning microbiota-targeted interventions as emerging modulators of stress-related risk trajectories. In parallel, machine-learning approaches now enable integrative interrogation of multi-omics datasets (genomics, proteomics, microbiome, neuroimaging, behavior), supporting finer stratification of risk and resilience and improving mapping between peripheral signatures and brain-behavior phenotypes (Nguyen et al., 2024; Lo-Ciganic et al.; Jadhav et al., 2022).

### 1.1 Literature search strategy

Relevant literature was identified through structured searches of PubMed/MEDLINE, Embase, and Web of Science. Searches primarily covered publications from 2000 to 2025; earlier seminal studies were included when foundational or mechanistically informative. Search strategies combined developmental, addiction, neuroimmune, and microbiome constructs using Boolean operators and, where applicable, controlled vocabulary. Representative queries included: (“adolescent∗” OR “development∗”) AND (“alcohol” OR “ethanol”) AND (“stress” OR “adversity”); (“microglia” OR “neuroinflammation∗” OR “cytokine∗”) AND (“addiction” OR “alcohol use disorder” OR “vulnerability”); (“gut microbiota” OR “microbiome” OR “intestinal permeability”) AND (“alcohol”) AND (“adolescent∗” OR “early-life stress”); and (“early-life stress” OR “adversity”) AND (“HPA axis” OR “glucocorticoid∗”) AND (“substance use” OR “alcohol”). Reference lists of key primary studies and selected comprehensive reviews were additionally screened to identify relevant articles not captured by database searches. Particular emphasis was placed on studies explicitly examining adolescence or developmental windows. Priority was given to longitudinal human cohorts, translational preclinical models, and mechanistic studies of neuroimmune signaling relevant to affective and executive circuit maturation. Adult clinical studies were included when mechanistically informative but are interpreted cautiously when extrapolated to adolescent-onset trajectories.

## 2 Clinical burden and diagnostic considerations

Adolescence, broadly spanning ages 10 to 24, represents a prolonged and sensitive developmental window during which vulnerability to psychiatric disorders and substance-use trajectories is disproportionately concentrated. This period carries substantial public health consequences: alcohol use disorder (AUD) ranks among the leading global causes of mortality and disability, claiming over three million lives and accounting for more than 130 million disability-adjusted life years annually (representing 5.1% of the global burden of disease and injury) with a disproportionate share of this burden falling on adolescents and young adults (Global status report on alcohol; Global and regional). Epidemiological studies indicate that approximately 20% of adolescents report patterns of heavy or binge drinking, and longitudinal neurocognitive research demonstrates that even sub-diagnostic levels of alcohol use are associated with impairments in inhibitory control and emotional regulation, preceding the fulfillment of formal diagnostic criteria (Sullivan et al., 2016).

First alcohol exposure, subsequent escalation, and the emergence of persistent use patterns tend to cluster within this developmental window, and exposure to stress or adversity during these years is robustly associated with earlier initiation and more rapid progression toward problematic use. The transition from experimental to compulsive consumption unfolds along a developmental trajectory characterized by progressive erosion of voluntary control over intake, whereby alcohol use persists despite escalating negative consequences. In the absence of early intervention, many at-risk individuals advance to AUD, a disorder affecting approximately 5% of the global population and marked by poor therapeutic responsiveness (Grant et al., 2015). The hallmark clinical feature of this progression is loss of control, manifesting as intolerance to delayed gratification, persistent craving, escalating compulsive use despite adverse outcomes, and reliance on alcohol for affective regulation (Leeman et al., 2012). These symptoms reliably predict chronicity and relapse vulnerability, establishing loss of control not merely as a diagnostic criterion but as a transdiagnostic dimension linking impulsive, compulsive, and affective domains. These converging clinical and epidemiological observations position adolescence as the critical developmental window during which stress-induced neuroimmune adaptations may become biologically embedded and subsequently expressed as impaired control over alcohol consumption (Crews and Vetreno, 2014). Peripheral systems such as the gut microbiota are accordingly conceptualized not as primary determinants of compulsive use, but as context-dependent modulators capable of amplifying neurobiological vulnerability under conditions of systemic strain.

Mechanistically, adolescence represents a period of heightened neuroplasticity coinciding with incomplete maturation of prefrontal regulatory circuits (Gogtay et al., 2004). During this developmental window, stress responsivity peaks while inhibitory control systems remain incompletely consolidated, creating conditions of enhanced vulnerability to environmental perturbations. Stressful life experiences during this time, may not only exacerbate impulsive behaviors (Wasserman et al., 2023) but have been linked to the onset of various psychiatric conditions (March-Llanes et al., 2017), including substance use disorders (SUDs). Indeed, early adversity, encompassing emotional neglect, caregiving instability, and socioeconomic deprivation, consistently emerges as the strongest predictor of AUD risk, with cumulative exposure burden demonstrating dose-response relationships with both onset timing and progression velocity (Sinha, 2024). The unpredictability and uncontrollability inherent in adversity exposures appear particularly damaging, establishing persistent alterations in stress-responsive circuits that bias individuals toward hypervigilance and maladaptive coping strategies (Heim and Nemeroff, 2001; Gee et al., 2013). Within this altered developmental landscape, alcohol acquires disproportionate salience as a means of affective regulation, explaining why early adversity not only predicts earlier initiation but accelerates the transition to problematic use patterns.

Clinically, this creates a predictable mismatch: most medications are calibrated on adult AUD chronicity, whereas adolescent-onset risk is often a moving target shaped by stress and plasticity, so timing and phenotype matter at least as much as the molecule. Pharmacotherapies such as naltrexone, acamprosate, and disulfiram show modest efficacy in adults (McPheeters et al., 2023) but are infrequently used in adolescents due to limited pediatric evidence and developmental safety concerns, when considered, they are typically reserved for selected cases and implemented with caution during ongoing brain maturation.

### 2.1 Limitations of traditional approaches

Historically, addiction research has emphasized organ-specific mechanisms, focusing predominantly on dopaminergic reward circuits and their modulation by drugs of abuse. These traditional models of addiction illuminated the neural basis of reinforcement and motivation but failed to account for the chronicity, relapse, and developmental specificity that characterize adolescent-onset AUD (Volkow et al., 1997). The exclusive focus on neural reward pathways fails to explain why vulnerability persists long after acute intoxication effects subside, and why conventional treatments targeting these circuits demonstrate limited efficacy (Anton et al., 2006). Contemporary systems biology approaches have begun to reveal addiction as a multisystem disorder involving dynamic interactions between central nervous system processes and peripheral inflammatory, metabolic, and endocrine pathways. This paradigm shift extends beyond traditional brain-centric models, recognizing that vulnerability to addictive behaviors emerges from interconnected biological networks rather than isolated reward circuits. And although central neuroimmune and affective circuits constitute the primary substrates of vulnerability, peripheral systems exert critical modulatory effects on risk trajectories rather than serving as mere epiphenomena. Among these, the gut microbiota has emerged as a biologically credible amplifier of central susceptibility (Cryan and Dinan, 2012), with preclinical studies revealing that early-life adversity could durably shift microbial ecology and gut-immune signaling (De Palma et al., 2015), and even alter the trajectory of microbiome recovery after stress cessation (Xu et al., 2020). Therefore, stress-induced dysbiosis may perpetuate low-grade systemic inflammation, perturb Hypothalamic Pituitary Adrenal (HPA) axis regulation, and skew affective processing toward negative valence. Mechanistic preclinical models implicate increased intestinal permeability, lipopolysaccharide translocation, and downstream cytokine cascades as convergent pathways through which peripheral instability reinforces central fragility (Leclercq et al., 2012, 2014). In humans, evidence remains largely correlative and highly context-dependent, with diet, early-life exposures, and comorbid conditions shaping observed associations (Leclercq et al., 2014). Consequently, the gut-brain axis should be regarded not as a primary driver of compulsive alcohol use, but as a contextual amplifier that magnifies preexisting central vulnerabilities under conditions of systemic strain. In adolescents already sensitized by stress, immune priming, or regulatory immaturity, dysbiosis may bias coping toward alcohol use. Clinically, this proportional framework is essential, microbiota-targeted interventions may offer adjunctive benefit by attenuating maladaptive inflammatory load and buffering stress responsivity (De Palma et al., 2015; Segovia-Rodríguez et al., 2022), yet they cannot supplant strategies that directly engage the neural mechanisms linking stress, immunity, and neurodevelopment. Their optimal application lies in augmenting multimodal paradigms that preserve neural plasticity and fortify regulatory control, rather than functioning as stand-alone therapies.

## 3 The neuroimmune framework

Recent advances in neuroimmunology have implicated neuroinflammation as a critical mediator linking early-life stress to persistent addiction vulnerability both in clinical and preclinical studies (Kirsch et al., 2024; Battista et al., 2023; Bekhbat et al., 2021; Carbia et al., 2021). Accordingly, the neuroimmune literature on adolescent alcohol vulnerability has moved beyond viewing inflammation as a simple downstream consequence of heavy drinking. Instead, inflammatory signaling is increasingly recognized as part of the mechanism through which stress and alcohol exposure become biologically embedded in the developing brain. Viewed across predominantly preclinical developmental work, four principles consistently emerge. First, adolescence is not simply a scaled-down adult state but a developmental interval in which immune signaling, glial activity, and synaptic refinement remain highly dynamic, rendering prefrontal-limbic circuitry particularly sensitive to environmental perturbation (Doremus-Fitzwater and Deak, 2022; Schalbetter et al., 2022). Second, alcohol exposure during this period does not merely provoke transient inflammatory activation, it can induce persistent microglial and broader glial remodeling that reshapes stress responsivity, synaptic maturation, and behavioral regulation well into adulthood (Melbourne et al., 2021; Vetreno et al., 2014). Third, neuroimmune signaling sits at a critical interface between developmental plasticity and addiction biology (Bekhbat et al., 2021), where inflammatory mediators can destabilize prefrontal-striatal-limbic circuits governing affect regulation, cognitive control, and reinforcement learning (Crews et al., 2023). Finally, this vulnerability is not restricted to the brain itself. Peripheral immune signals, particularly those arising from gut-microbiota dysregulation and stress-related increases in intestinal permeability, can amplify central inflammatory tone and further bias developing regulatory circuits toward maladaptive persistence (Xu et al., 2020; Carbia et al., 2021). What remains unresolved is how transient neuroimmune perturbations during adolescence become stabilized as enduring features of circuit organization

Because immune signaling and synaptic refinement remain tightly coupled during this developmental interval, stress or alcohol exposure has the potential to recalibrate glial regulation of circuit maturation, redirecting the trajectory of regulatory network development. Evidence from preclinical models indicates that stress exposure during adolescence, when immune-to-brain signaling is tightly coupled to ongoing circuit maturation, can produce long-lasting effects that persist into adulthood and shape inflammatory responses to subsequent challenges. In these models, chronic adversity is associated with lasting microglial priming and establishes a state of latent immune hyperreactivity that may persist into adulthood (Frank et al., 2012; Bolton et al., 2022; Reemst et al., 2022; Vetreno et al., 2018). Upon subsequent challenge, including stress or alcohol exposure, these primed immune cells release exaggerated quantities of pro-inflammatory cytokines, including interleukin-6 (IL-6), tumor necrosis factor-α (TNF-α), and interleukin-1β (IL-1β) (Bekhbat et al., 2021; Frank et al., 2012; Weber et al., 2015; Lippai et al., 2013). This inflammatory cascade is hypothesized to disrupt synaptic plasticity within prefrontal-striatal-limbic networks governing executive control, emotional regulation, and salience processing. As these circuits lose adaptive capacity, behavior may shift toward disinhibition and affect-driven responding, thereby establishing the neurobiological substrate for compulsive alcohol use (Crews et al., 2016; Varodayan et al., 2023).

**Fig. 1:**
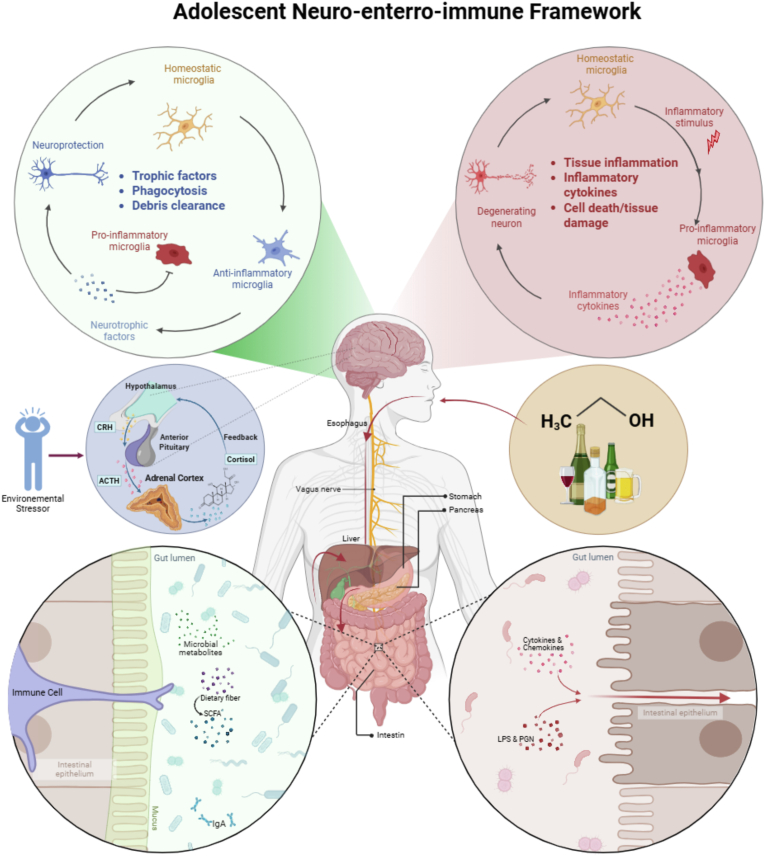
Adolescent neuro-entero-immune framework linking stress, alcohol exposure, and neuroimmune dysregulation. This figure presents a proposed adolescent neuro-entero-immune framework illustrating how environmental stress and alcohol exposure converge across gut, peripheral, and central nervous system pathways to shape neurobiological vulnerability during adolescence. Environmental stressors activate the hypothalamic-pituitary-adrenal (HPA) axis, driving cortisol release with downstream consequences for immune regulation, microbial ecology, and neural function. In parallel, alcohol exerts direct toxic effects on multiple gastrointestinal organs (including the esophagus, stomach, intestine, liver, and pancreas) as well as on the brain itself. Within the gut, stress- and alcohol-related perturbations may disrupt microbial homeostasis, deplete beneficial microbial metabolites such as short-chain fatty acids (SCFAs), and compromise intestinal epithelial barrier integrity. This barrier disruption facilitates the translocation of microbial products (including lipopolysaccharide (LPS) and peptidoglycan (PGN)) into systemic circulation, amplifying peripheral cytokine and chemokine signaling. These peripheral inflammatory signals may reach the brain via humoral, neural, and cellular gut-brain communication pathways, contributing to central neuroimmune activation. The left panel depicts a homeostatic microglial state, in which balanced glial activity supports neuroprotection through trophic factor release, phagocytosis of cellular debris, and activity-dependent synaptic pruning. The right panel illustrates a neuroinflammatory state, characterized by pro-inflammatory microglial activation, cytokine release, tissue inflammation, and neuronal damage. Together, these interactions illustrate how the convergence of stress and alcohol exposure during adolescence may promote sustained gut-brain axis dysregulation and central neuroimmune vulnerability. For clarity, immune responses within the enteric nervous system (ENS) are not depicted in this schematic.

Importantly, neuroimmune mechanisms should be framed not as an alternative to classical neurobiological models of addiction, but as a modulatory layer that shapes how circuit adaptations are initiated, expressed, and maintained across development. Cytokine signaling and microglial reactivity can bias excitatory-inhibitory balance, alter glutamatergic plasticity, and influence dopaminergic prediction-error and salience processing, thereby shifting reinforcement learning toward habit-like control (Varodayan et al., 2023; Treadway et al., 2017). In parallel, immune-driven effects on stress circuitry (including HPA-axis calibration and CRF-related systems) can intensify negative affect and withdrawal-associated dysphoria, strengthening negative reinforcement processes (Bajo et al., 2008). Together, these interactions position neuroinflammation as a key amplifier within prefrontal-striatal-limbic networks that support escalation, loss of control, and relapse vulnerability (see Fig. 1).

### 3.1 Stress-induced neuroendocrine-neuroimmune activation

The HPA-axis demonstrates heightened reactivity during adolescence, reflecting incomplete maturation of glucocorticoid feedback mechanisms. Under conditions of chronic or repeated stress exposure, this system becomes persistently activated, producing sustained cortisol elevations that alter stress responsivity calibration and compromise homeostatic regulation (McEwen, 1998; Gunnar et al., 2009). Prolonged HPA activation during adolescence particularly impacts the prefrontal cortex (PFC), which serves as the primary regulator of executive control and inhibitory function. Chronic glucocorticoid exposure compromises PFC integration with motivational and affective networks, delaying maturation of top-down regulatory pathways while simultaneously enhancing limbic reactivity (Gee et al., 2013; Tottenham and Galván, 2016). This creates a fundamental imbalance wherein heightened emotional drive occurs in the context of immature prefrontal oversight (Gee et al., 2013), constituting a core pathway through which stress fosters maladaptive coping patterns (Bekhbat et al., 2021). Importantly, these alterations rarely normalize following stress cessation. Instead, they recalibrate regulatory circuits around chronically elevated allostatic setpoints, manifesting clinically as emotional lability, reduced distress tolerance, and diminished behavioral flexibility.

In parallel, stress-related endocrine signals interface directly with neuroimmune regulation. Early adversity induces persistent priming of microglia and astrocytes, the brain's resident immune cells, establishing a state of enhanced inflammatory reactivity that persists long after initial stress exposure. In developmental models, this priming phenomenon is not constrained to mere activation intensity, early-life stress can perturb microglial maturation and homeostatic functions, including activity-dependent synaptic pruning within stress-regulatory networks (for example CRH-expressing neuronal populations), thereby coupling immune dysregulation to circuit remodeling (Bolton et al., 2022; Reemst et al., 2022; Delpech et al., 2016). Mechanistically, priming has been linked to epigenetic modifications that lower activation thresholds and amplify cytokine responses to subsequent challenges (Frank et al., 2012). Upon re-exposure to stress or alcohol, danger-associated signaling, in particular HMGB1, can act as a priming stimulus engaging NLRP3 inflammasome pathways and biasing microglia toward exaggerated pro-inflammatory induction (Weber et al., 2015; Vetreno and Crews, 2012). In parallel, ethanol exposure itself can amplify neuroinflammation, via NLRP3/ASC-dependent IL-1β signaling providing a tractable route by which alcohol functions as a second hit on a sensitized neuroimmune background (Treadway et al., 2017). Downstream, cytokines function beyond peripheral immunological markers, they can act as neuromodulatory signals that tune addiction-relevant circuit computations. In preclinical rodent models, chronic ethanol shifts IL-1β regulation of GABAergic transmission toward a pro-inflammatory mode that can weaken inhibitory control (Varodayan et al., 2023). In adult humans studies, IL-6 has been associated with stress-related modulation of striatal prediction-error signals, linking inflammatory tone to reinforcement-learning processes, notably alteration of salience attribution (Treadway et al., 2017). This supports a model where inflammatory response extends beyond acute activation phases, establishing a state of chronic low-grade neuroinflammation that maintains a permissive environment for ongoing circuit dysfunction (Vetreno et al., 2017). Consistent with this, human studies report that peripheral inflammatory markers, including C-reactive protein (CRP) and circulating cytokines, are altered in AUD and SUDs and relate to affective symptom burden (Martinez et al., 2018). Stress-linked cytokine dynamics have also been shown to prospectively predict relapse in alcohol-dependent individuals (Fox et al., 2020).

Taken together, these endocrine-immune interactions do not translate into a diffuse inflammatory fog, they bias specific developing control circuits that are still under construction in adolescence. Neuroinflammation demonstrates regional selectivity for circuits implicated in addiction vulnerability. Preclinical models indicate that cytokine signaling within the medial prefrontal cortex disrupts excitatory-inhibitory balance and impairs top-down control over limbic and striatal targets, effectively taking the foot off a prefrontal brake that is still being tuned and consolidated across adolescence (Varodayan et al., 2023). Once that brake slips, the system doesn't simply become more emotional, it becomes more cue-driven, as bottom-up signals face less cortical gating. Inflammatory mediators also compromise white matter integrity and myelination, weakening long-range connectivity essential for executive function. Human neuroimaging studies in adults suggest that inflammatory signaling interacts with dopaminergic prediction-error processes within striatal regions (Treadway et al., 2017), biasing dopamine-glutamate interactions toward habit formation while reducing cognitive flexibility (Tricomi et al., 2009). Consequently, this shift accelerates the transition from goal-directed to stimulus-response control, functionally locking in short-horizon solutions when uncertainty and stress are high. Consistent with this circuit logic, the anterior cingulate cortex, crucial for conflict monitoring and decision-making, also shows enhanced inflammatory sensitivity that correlates with impulsivity and poor treatment outcomes in adults with SUDs (Fox et al., 2020). Amygdalar neuroinflammation heightens threat detection and emotional reactivity while impairing fear extinction, contributing to persistent anxiety and negative affect that drive continued substance use (Bajo et al., 2008). Although most mechanistic evidence derives from preclinical models or adult human studies, these circuit-biased effects are likely to be amplified in adolescent-onset trajectories, when regulatory connectivity and myelination are still consolidating and therefore more susceptible to immune-mediated tuning (Gee et al., 2013). These circuit-specific effects collectively create a neurobiological profile characterized by enhanced stress reactivity, diminished cognitive control, and increased reliance on external regulation.

### 3.2 Gut microbiota-immune-brain axis and the shaping of drug addiction

While central neuroimmune and affective circuits constitute the primary determinants of vulnerability, peripheral modulators exert decisive influences on risk trajectories rather than serving merely as downstream consequences. Among these, the gut microbiota has emerged as a mechanistically plausible amplifier of central susceptibility (De Palma et al., 2015; Leclercq et al., 2012, 2014; Segovia-Rodríguez et al., 2022). Adolescence represents a sensitive window of plasticity for this amplification, as stress exposure can concurrently shape microbial ecology trajectory and immune signaling during ongoing stress-regulatory systems maturation (Xu et al., 2020; Macht et al., 2020; Dunphy-Doherty et al., 2018). Evidence for developmental specificity is now strongest in preclinical adolescent models. Chronic stress alters microbiome recovery trajectories and microbial metabolite profiles after stress cessation (Xu et al., 2020), and adolescent intermittent ethanol exposure produces long-lasting dysbiosis with altered enteric neurochemical signaling that persists into adulthood (Vetreno et al., 2021). In contrast, human evidence is derived mainly from adult AUD cohorts and remains more limited and largely associative not causal. Stress-induced dysbiosis may sustain chronic low-grade inflammation, disrupt HPA-axis regulation, and biases affective processing toward negative valence (De Palma et al., 2015; Crumeyrolle-Arias et al., 2014). Clinical investigations in adults with AUD have identified associations between enhanced intestinal permeability, lipopolysaccharide translocation, and downstream cytokine signaling, implicating them as convergent pathways through which peripheral dysfunction could reinforces central vulnerability (Leclercq et al., 2012, 2014). However, these associations appear highly sensitive to contextual moderators, including diet, metabolic health, medication exposure, sleep disruption, psychiatric comorbidity, and socioeconomic adversity. Heterogeneous sampling and predominantly cross-sectional designs further constrain causal inference, and microbiota signatures are not uniform across individuals with AUD. Consequently, the gut-brain axis should be conceptualized not as a primary etiological factor in compulsive alcohol use, but as a contextual modulator that amplifies preexisting central vulnerabilities under conditions of elevated systemic burden (see Fig. 1). In this developmental context, gut-derived inflammatory signaling may further amplify central immune priming (including microglial sensitization), thereby lowering the threshold for alcohol use as a compensatory regulatory behavior (Xu et al., 2020; Segovia-Rodríguez et al., 2022). Clinically, this proportional framework (Carbia et al., 2021) is critical, microbiota-targeted interventions may provide adjunctive therapeutic benefit through attenuation of inflammatory burden and enhancement of stress buffering capacity, yet they cannot replace strategies that directly engage the neural substrates linking stress, immune activation, and neurodevelopmental processes. Their optimal utility resides in augmenting multimodal treatment paradigms designed to preserve neural plasticity and strengthen regulatory control, rather than functioning as monotherapeutic approaches.

## 4 Alcohol use and self-medication theory

### 4.1 Intrinsic neurobiological effects of alcohol

Alcohol drinking has long been shown to exert direct effects on the brain. In particular, early alcohol exposure has been shown to correlate with disruption of the developmental trajectories underlying inhibitory control, affect regulation and reward processing in animal models and human neuroimaging cohorts (Crews et al., 2016; Squeglia et al., 2015). Acutely, alcohol acts as a positive allosteric modulator at GABA-A receptors while antagonizing glutamatergic NMDA receptor signaling, producing fast shifts in the excitatory-inhibitory balance that reduce transiently anxiety while impairing cognitive control and learning processes (Field et al., 2010). Preclinical studies have demonstrated that repeated exposure during a highly plastic developmental window disrupts normative myelination processes and synaptic pruning, inducing longer-lasting adaptations in GABAergic and glutamatergic signaling that contribute to tolerance, withdrawal-associated hyperexcitability, and impaired regulatory control (Crews et al., 2016; Kroener et al., 2012; Roberto et al., 2004). In parallel, alcohol directly modulates neuromodulatory systems essential for motivation and affect regulation. Adult human PET studies indicate that ethanol alters mesolimbic dopaminergic signaling and reinforcement learning dynamics (Volkow et al., 2007; Yoder et al., 2007), while rodent studies further implicate modulation of serotonergic tone, and recruitment of stress-related peptidergic systems, including endogenous opioid, neuropeptide Y, and corticotropin-releasing factor signaling pathways (Funk et al., 2006; Thiele et al., 1998). Collectively, these mechanisms may contribute to alterations in reward salience, stress responsivity, and emotion regulation during ongoing circuit maturation. Importantly, these intrinsic neurobiological effects are unlikely to operate independently of the neuroimmune milieu. Alcohol's direct neuromodulatory actions may interact with stress-related immune priming, and together these processes can bias the maturation of regulatory circuitry toward heightened reactivity and weaker top-down control. Thus, neuroinflammation should be conceptualized as one convergent pathway that can magnify risk during this developmental window. Within this framework, it is important to distinguish adaptive immune signaling from pathological neuroinflammation. Under physiological conditions, transient inflammatory responses and basal microglial activity support host defense, tissue repair, and experience-dependent synaptic pruning. Neurobiological vulnerability during adolescence is therefore unlikely to arise from immune activation per se, but rather from the persistence, amplification, or developmental mistiming of inflammatory signaling, which can constrain regulatory flexibility. Accordingly, the therapeutic objective is not broad immune suppression but the resolution of pathological hyperreactivity and restoration of neuroimmune homeostasis.

### 4.2 Anxiolytic effects and emotional relief

Preclinical studies have demonstrated that alcohol's rapid anxiolytic properties result from its potentiation of GABAergic transmission and attenuation of stress-reactive circuit activity, particularly within the amygdala (Bajo et al., 2008). In adolescent models with stress-sensitized inflammatory profiles, this anxiolytic effect acquires disproportionate motivational significance (Vore et al., 2017; Varlinskaya and Spear, 2006; Chaby et al., 2015). The contrast between internal tension and alcohol-induced relief creates a powerful learning signal that is encoded with exceptional strength (Wyvell and Berridge, 2001). This pharmacological relief functions as a substitute for deficient endogenous regulatory capacity. Adolescents exposed to chronic adversity often lack reliable frameworks for developing autonomous emotion regulation skills. Within this regulatory void, alcohol could serve as an external modulatory agent, providing immediate, though temporary, compensation for immature or compromised internal mechanisms (Khantzian, 1985; Shadur et al., 2015). The functional nature of this relief is critical to understanding addiction development. Alcohol use is reinforced not solely through hedonic mechanisms but through its capacity to restore emotional equilibrium, establishing its role as an adaptive coping strategy within the individual's behavioral repertoire (Koob and Moal, 1997; Funk et al., 2006; Valdez et al., 2003).

### 4.3 Enhanced reward salience, learning, and disinhibition

Chronic stress in rodents sensitizes mesolimbic dopamine pathways, amplifying the salience of rewarding stimuli including alcohol (Abercrombie et al., 1989; Kabbaj and Isgor, 2007). Human translational studies indicate that acute stress-induced increases in IL-6 alter dopaminergic prediction-error signaling within the adult striatum, shifting how reward cues are processed (Treadway et al., 2017). When interpreted within a developmental context, this neuroimmune crosstalk offers a compelling mechanism for the enhanced reactivity to both stress and reward cues that uniquely primes adolescents for addiction. For stress-exposed adolescents, alcohol becomes more than a hedonic reinforcer, it functions as a regulatory anchor that provides predictable relief from internal instability. Each successful use episode strengthens the association between consumption and relief, progressively consolidating alcohol's position within the individual's coping repertoire while narrowing behavioral alternatives. Inflammatory priming also enhances cue-reward learning while impairing extinction processes (Macht et al., 2020; Montesinos et al., 2016). Environmental stimuli associated with alcohol acquire lasting motivational properties that persist long after consumption episodes, creating a neurobiological substrate for relapse vulnerability. Alcohol's acute effects extend beyond anxiolysis to include suppression of prefrontal cortical activity, particularly in regions mediating cognitive control and conflict monitoring. For adolescents with stress-compromised prefrontal function, this effect provides subjective relief from internal tension and competing motivational demands. However, this cognitive suppression carries significant costs. By weakening executive control mechanisms (Field et al., 2010), alcohol consumption renders impulsive responses more likely while simultaneously reducing awareness of negative consequences. What initially provides temporary relief from psychological conflict progressively erodes the neural capacity for self-regulation. This creates a paradox whereby alcohol use is adopted for transient, perceived relief, yet repeated alcohol exposure further destabilizes the very circuits mediating affect regulation and inhibitory control (Kroener et al., 2012; Koob, 2017). Each use episode reinforces the perception of alcohol as an effective coping strategy while deepening the vulnerability it ostensibly addresses (Roberto et al., 2004).

## 5 Withdrawal and relapse dynamics

Alcohol withdrawal unmasks the underlying dysregulation established by chronic stress and inflammatory priming (Vore et al., 2017; Pascual et al., 2007). In adolescent rodent models, intermittent ethanol exposure leaves an enduring neuroimmune imprint (Li et al., 2025) and, in paradigms probing endocrine outcomes, a persistent recalibration of HPA-axis responsivity (Vore et al., 2017). Critically, these liabilities are often most apparent during early abstinence, consistent with the interpretation that withdrawal reveals latent dysregulation rather than generating it de novo (Li et al., 2025). Paralleling these findings in adult clinical cohorts, alcohol cessation is likewise coupled to measurable stress-system disruption and negative affect, including altered HPA-axis function during early abstinence (Adinoff et al., 1990) and withdrawal-associated network changes that track impaired emotional regulation (O'Daly et al., 2012). Indeed, in individuals who have relied on alcohol for regulatory purposes, cessation removes the pharmacological constraint on hyperactive stress circuits, exposing a system deprived of its external buffer. The resulting affective destabilization manifests as intense irritability, anxiety, and emotional volatility that may represent not transient withdrawal discomfort but the unmasking of a chronically dysregulated system. HPA-axis hyperactivity during withdrawal produces pathologically sustained cortisol elevations, particularly in previously stress-sensitized individuals (Adinoff et al., 1990). A critical developmental caveat is that human adolescent endocrine data remain limited, however, developmental stress models suggest that comparable rebound dynamics may be amplified when withdrawal occurs on a background of immature feedback regulation. This endocrine rebound creates a state of chronic internal alarm characterized by heightened vigilance, negative affect amplification, and catastrophic interpretation of minor stressors. Rather than facilitating recovery, abstinence is frequently associated with a period of maximal vulnerability wherein even minimal perturbations are experienced as overwhelming (O'Daly et al., 2012).

In parallel, withdrawal-related immune activation is not confined to the CNS but is accompanied by measurable peripheral inflammatory signals. In adult AUD cohorts, altered levels of systemic inflammatory markers, including IL-6, CRP, and indices of endotoxin exposure consistent with gut-derived LPS translocation, have been reported, particularly around periods of heavy use and abstinence (Leclercq et al., 2014; Heberlein et al., 2014). These inflammatory mediators are likely to drive a cascade of somatic complaints including sleep disruption (Brower et al., 1998), gastrointestinal distress (Leclercq et al., 2014), fatigue, and malaise. Importantly, these symptoms are not merely correlates of withdrawal but may constitute active biological drivers of distress that fuel relapse vulnerability. The convergence of affective and somatic suffering creates a unified destabilizing state wherein each domain amplifies the other. Physical discomfort intensifies emotional distress while psychological volatility magnifies the subjective impact of bodily symptoms, creating a self-reinforcing cycle of escalating discomfort.

Within this context, relapse may function as short-term allostatic relief rather than a simple failure of control. By acutely dampening stress reactivity and dampening immune hyperactivity (Afshar et al., 2015), alcohol consumption temporarily restores a state of precarious but familiar equilibrium. In adolescents, this relief is acquired while regulatory circuits remain under consolidation, potentially strengthening learning signals that couple negative affect to alcohol seeking. In contrast, adult relapses may more often reflect reinstatement of established habits on a background of more stable circuit organization**.** For the stress-sensitized adolescent, in the context of ongoing neurodevelopmental maturation, drinking is encoded not as pleasure-seeking but as restoration of internal balance that abstinence has destabilized (Squeglia et al., 2015; Khantzian, 1985). This reframes relapse from moral failure to predictable biological response, wherein the system attempts to reinstate its accustomed regulatory configuration. Each relapse episode, however, further entrenches the underlying pathophysiology. Stress responsivity is pushed deeper into dysregulation, inflammatory priming becomes more thoroughly embedded, and neural substrates of self-regulation suffer progressive erosion. What begins as adaptive recalibration evolves into rigid maladaptation resistant to intervention (Gass et al., 2014).

## 6 Structural consolidation and chronicity

Chronic neuroinflammation transforms initially reversible alterations into stable structural constraints through several converging mechanisms (Erickson et al., 2019). Preclinical studies have shown that sustained cytokine signaling is associated with impairment in oligodendrocyte function and in myelination (Favrais et al., 2011). Consistent with these cellular mechanisms, human neuroimaging studies in adolescents confirm that elevated peripheral inflammatory markers correlate with reduced white matter integrity, effectively weakening long-range prefrontal control over limbic and striatal targets (Li et al., 2025). Blood-brain barrier permeability increases under inflammatory pressure, facilitating continued immune activation and perpetuating central dysregulation (Togre et al.; Lowe et al., 2018). These processes embed stress biology into neural architecture, converting adaptive allostatic responses into fixed structural configurations. Neuroimaging studies in stress-exposed adolescents with escalating alcohol use reveal persistent prefrontal-amygdala disconnection, exaggerated salience network reactivity, and reduced cognitive control capacity that persists even during abstinent periods (Jacobson et al., 2023; Whelan et al., 2014). Peripheral inflammatory markers mirror these central changes, with elevated IL-6, CRP, and TNF-α predicting poor treatment response and accelerated relapse (Heberlein et al., 2014). The correlation between inflammatory burden and addiction severity most likely underscores neuroinflammation's role not as epiphenomenon but as active driver of disease progression.

### 6.1 Habit formation, plasticity closure and trait stabilization

As alcohol use becomes repetitive, its role transitions from intentional coping strategy to automatized behavioral routine (Giannone et al., 2024). Preclinical studies have demonstrated that stress-induced alterations in striatal learning mechanisms accelerate this transition by biasing dopaminergic signaling toward habit-promoting pathways while weakening goal-directed control systems (Dias-Ferreira et al., 2009). Neuroinflammation contributes to this process by compromising prefrontal regulation of cortico-striatal circuits and facilitating the emergence of stimulus-driven behaviors (Felger and Treadway, 2017). Environmental cues associated with alcohol acquire disproportionate control over behavior, triggering consumption responses that bypass reflective decision-making processes (Garbusow et al., 2016). This progression represents the displacement of flexible, goal-directed control by rigid, context-independent responding. Neuroimaging reveals dorsal striatal dominance during cue exposure, while peripheral inflammatory markers correlate with enhanced habit bias and reduced cognitive flexibility.

Once habit-like control becomes dominant, chronic low-grade neuroinflammation may further constrain the developmental plasticity that would otherwise support recovery. Microglial priming, astrocytic dysfunction, and persistent cytokine signaling maintain a neurobiological environment that favors maintenance of maladaptive configurations over adaptive reorganization (Erickson et al., 2019). Rather than supporting recovery, residual plasticity becomes redirected toward reinforcing dysfunctional patterns. Neural flexibility that might facilitate therapeutic change instead consolidates addiction-related learning and strengthens relapse-promoting associations (Lüscher et al., 2020). This transformation manifests clinically as the emergence of seemingly fixed personality traits including impulsivity, emotional instability, and poor executive control (Everitt and Robbins, 2016). What appears as constitutional vulnerability represents the end-product of immune-mediated remodeling, wherein modifiable risk factors have been biologically embedded and stabilized. Peripheral biomarkers reflect this consolidation process, with persistent elevation of inflammatory markers predicting treatment resistance and poor long-term outcomes. The apparent “hardwiring” of addiction vulnerability reflects not intrinsic pathology but the biological embedding of stress-induced alterations that have exceeded the window for spontaneous recovery (Koob and Schulkin, 2019).

## 7 Therapeutic implications

During mid-to-late adolescence, stress-regulatory and neuroimmune processes remain comparatively dynamic, such that risk trajectories may be more amenable to recalibration before they consolidate into rigid maladaptive patterns (Gogtay et al., 2004; Gunnar et al., 2009). This window is not developmentally uniform, relative to adults, adolescents exhibit ongoing maturation of regulatory circuitry and heightened sensitivity to environmental perturbation, which can amplify risk but also increase the leverage of early intervention (Gogtay et al., 2004; Gee et al., 2013; Bekhbat et al., 2021). In contrast, adult-onset trajectories unfold on a background of more consolidated circuitry and less flexible stress-regulatory dynamics, shifting the balance from prevention toward stabilization and relapse prevention. Clinically, early warning signals, including emotional volatility, impulsive coping, and initial alcohol use for anxiolysis, should prompt assessment and intervention rather than being dismissed as normative adolescent behavior. Once consolidation occurs, manifesting as persistent affective instability, cue-driven consumption patterns, and resistance to environmental contingencies, therapeutic leverage diminishes substantially across all intervention modalities (Volkow and Boyle, 2018; Sinha, 2011). The window for prevention-focused approaches closes rapidly, emphasizing the critical importance of early identification and proactive treatment.

### 7.1 Neuroimmune-modulatory therapeutic strategies

Maladaptive neuroimmune signaling is increasingly implicated in addiction vulnerability, supporting targeted neuroimmune modulation as a mechanistically grounded adjunctive therapeutic strategy. Human evidence, however, remains limited and heterogeneous. Microglia-linked immunomodulation (e.g., minocycline) has shown mixed but suggestive effects on drinking-related outcomes in heavy drinkers, although replication and integration of inflammatory biomarkers are required to establish target engagement and define responder profiles (Petrakis et al., 2019). Converging preclinical evidence supports this framework. Phosphodiesterase-4 inhibition (e.g., apremilast) reduces alcohol intake while modulating central amygdala neurophysiology, delineating a tractable pathway linking immune signaling to addiction-relevant circuit function. Clinical translation of these effects remains to be established. Incretin-based therapies have more recently advanced into clinical evaluation. Randomized trials provide initial evidence that GLP-1 receptor agonists reduce alcohol craving and/or intake in controlled settings and treatment-seeking samples, although effect sizes and endpoint specificity vary across studies (Chuong et al., 2023). Complementary pharmaco-epidemiological analyses extend these findings to real-world contexts, reporting associations between GLP-1 receptor agonist exposure and reduced alcohol consumption, without establishing causality. These findings highlight pathway-specific effects that may not generalize across incretin-modulating agents (Farokhnia et al., 2025). Taken together, these data support the need for biomarker-informed, adequately powered trials to resolve current heterogeneity and identify patient subgroups most likely to benefit from immune- and metabolic-targeted adjunctive interventions. In parallel, non-pharmacological interventions with established anti-inflammatory effects provide accessible adjunctive strategies. Structured physical activity, sleep optimization, circadian rhythm stabilization, and nutritional interventions demonstrate measurable reductions in inflammatory burden while supporting stress resilience (Vetreno et al., 2018; Liu et al., 2022; Irwin et al., 2015; Pauluci et al., 2022). These lifestyle modifications should not be viewed as secondary interventions but as essential components of comprehensive treatment approaches.

These observations further suggest that treatment response is unlikely to be uniform across individuals. Adolescents characterized by elevated inflammatory tone or evidence of neuroimmune sensitization may derive greater benefit from immune-targeted adjunctive strategies, whereas others may respond adequately to behavioral and environmental interventions. This underscores the need for stratified approaches aligned with individual biological profiles and developmental stage.

Consistent with this heterogeneity, effective intervention is unlikely to be achieved by targeting any single pathway in isolation. Within a neuroimmune framework, sustained benefit will likely require coordinated engagement of biological, behavioral, and environmental processes that jointly shape stress responsivity and inflammatory tone. Anti-inflammatory approaches may provide complementary benefit but should be integrated with interventions that strengthen regulatory capacity (e.g., emotion regulation and executive control) and reduce chronic stress burden. In adolescents, environmental stabilization, particularly reductions in unpredictability and sustained psychosocial stress, may be a key determinant of whether biological and behavioral gains are maintained over time. Accordingly, multimodal strategies that align physiological modulation with skills-based and contextual interventions are best aligned with the systems-level vulnerability profile outlined here.

## 8 Future directions and clinical translation

### 8.1 Biomarker-informed stratification and research priorities

The marked heterogeneity of adolescent vulnerability further supports the development of biomarker-informed stratification approaches. Peripheral inflammatory markers, stress responsivity indices, and circuit-level measures may help identify high-risk phenotypes and guide the timing and intensity of intervention. The neuroimmune framework suggests specific biomarker profiles that could enable early identification and risk stratification (Whelan et al., 2014). Peripheral inflammatory markers including IL-6, CRP, and TNF-α demonstrate predictive utility when combined with measures of autonomic dysfunction and neuroimaging indices of circuit connectivity. Advanced analytical approaches including machine learning algorithms show promise for integrating multi-dimensional datasets encompassing inflammatory, genetic, neuroimaging, and behavioral variables (Nguyen et al., 2024; Lo-Ciganic et al.; Jadhav et al., 2022). These approaches may enable personalized risk prediction and treatment selection based on individual inflammatory and neurodevelopmental profiles. Real-time monitoring technologies (Alinia et al., 2021; Bae et al., 2017) (via mobile or wearable devices) could facilitate continuous assessment of stress exposure, inflammatory responses, and behavioral changes, offering unprecedented opportunities for precision intervention. To translate these approaches, several methodological gaps require targeted study. Longitudinal studies tracking inflammatory trajectories from early adversity through addiction development are needed to identify critical transition points and intervention opportunities (Kautz et al., 2020). Mechanistic research using advanced neuroimaging and molecular techniques could clarify the causal relationships between specific inflammatory mediators and circuit dysfunction. Clinical trials of anti-inflammatory interventions in adolescent populations require careful attention to developmental considerations and safety profiles. Combination therapy approaches integrating anti-inflammatory, behavioral, and environmental interventions need systematic evaluation to optimize treatment protocols. The role of individual difference factors including sex, genetic background, and comorbid conditions in modulating inflammatory responses requires detailed investigation. These factors may significantly influence treatment response and necessitate further stratification of intervention approaches.

### 8.2 Translational perspective on gut-brain modulation

The gut-brain axis represents a biologically plausible pathway that contributes to adolescent vulnerability to alcohol misuse (Diaz et al., 2011). Emerging evidence positions the gut microbiota as a modifiable risk factor, particularly following early adversity where microbial alterations coincide with heightened stress reactivity and systemic inflammation (De Palma et al., 2015). Clinical studies demonstrate that microbiota alterations and increased intestinal permeability occur in only a subset of patients with AUD (Leclercq et al., 2012). However, when present, these changes strongly correlate with craving intensity, affective disturbance, and poorer treatment outcomes. Dysbiosis therefore functions not as a universal AUD hallmark, but as a phenotypic amplifier that accelerates severity trajectories. Interventions targeting microbial balance demonstrate measurable therapeutic effects. Probiotics, prebiotics, and dietary modifications reduce systemic inflammatory burden and improve barrier function in psychiatric and metabolic populations (Barrera-Conde et al., 2025; Irwin et al., 2018). Preliminary AUD trials report improvements in craving and affect regulation. A randomized trial of fecal microbiota transplantation in adults with AUD normalized barrier function and shifted circulating metabolites, though safety and durability in adolescents remain untested. Two translational principles emerge from this evidence: First, microbiota-targeted strategies prove most relevant for phenotypes characterized by systemic amplification. This includes adolescents with adversity-related dysbiosis, metabolic comorbidities, or biomarker evidence of heightened inflammation. In these contexts, gut-directed interventions can enhance pharmacological and behavioral treatment efficacy by reducing inflammatory drive and supporting resilience.

Second, efficacy depends critically on developmental timing. During adolescence, when microbiota composition, immune tone, endocrine feedback, and prefrontal-limbic connectivity remain plastic, modulation can redirect trajectories toward recovery. Once maladaptive circuits consolidate, peripheral interventions alone cannot reverse entrenched compulsivity.

Clinically, the gut-brain axis should be regarded neither as a panacea nor as irrelevant. Its role is adjunctive: insufficient in isolation, but capable of amplifying multimodal interventions when applied within a stratified framework. Anchoring its use in phenotype, timing, and context offers the most pragmatic translation route. In this calibrated role, gut-brain axis modulation emerges as a biologically plausible amplifier that broadens central therapy impact by dampening peripheral inflammatory drivers of vulnerability during this uniquely malleable developmental window. These strategies underscore that preventing compulsive alcohol use requires multidimensional and time-sensitive intervention. Adolescence offers a narrow but decisive window during which stress-driven neuroimmune remodeling remains dynamic and behavior remains malleable. By combining pharmacological tools that normalize maladaptive immune hyperreactivity, behavioral therapies that strengthen executive control, ecological stabilization that reduces chronic stress exposure, and microbiota-directed interventions in stratified contexts, clinicians can preserve developmental flexibility and prevent loss of control from becoming a fixed phenotype.

## 9 Conclusion

**Fig. 2:**
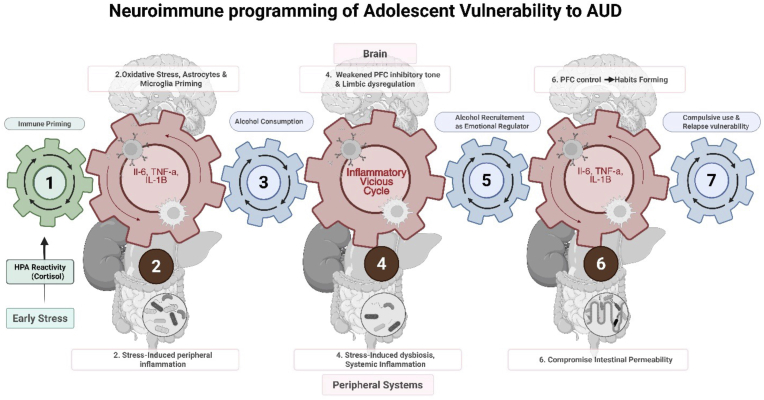
Early-life stress initiates a self-perpetuating neuro-immune–behavioral cycle promoting maladaptive alcohol consumption. Early-life adversities including housing instability, psychosocial trauma, or physical stress amplify hypothalamic-pituitary-adrenal (HPA) axis reactivity and prime systemic innate immune responses (1). Sustained elevation of glucocorticoid and pro-inflammatory cytokine signaling (interleukin-1β, interleukin-6, tumor necrosis factor-α) is associated with microglial and astrocytic activation, mitochondrial oxidative stress, and peripheral immunological priming (2). Initial alcohol use is introduced against this background of neuroimmune sensitization, entering a system already primed for heightened reactivity (3). Within this sensitized milieu, the combined effects of stress exposure and early alcohol use destabilize gut microbial communities and compromise intestinal barrier function, allowing lipopolysaccharide to enter systemic circulation and sustain toll-like receptor–mediated neuroinflammatory signaling, while gradually weakening prefrontal GABAergic inhibitory control over limbic reward and stress-response circuits (4). These neuroimmune alterations reduce cognitive and emotional regulation capacity, promoting alcohol consumption as a putative negative reinforcer and emotion-regulation strategy. Chronic alcohol exposure further compromises intestinal barrier integrity, exacerbates bacterial translocation and systemic endotoxemia, and intensifies central neuroinflammation. (5) These alcohol-driven alterations potentiate prefrontal disinhibition and dorsolateral striatal habit learning, (6,7) culminating in a consolidated behavioral phenotype characterized by compulsive consumption patterns and heightened relapse susceptibility despite adverse consequences.

Adolescent vulnerability to alcohol use disorder emerges through stress-induced neuroimmune recalibration rather than isolated neural dysfunction. Early adversity establishes chronic inflammatory priming that disrupts developing regulatory circuits while simultaneously creating conditions wherein alcohol acquires disproportionate utility as an external regulatory agent. The resulting dependence relationship becomes progressively entrenched through inflammatory-mediated consolidation processes that transform initially reversible vulnerabilities into stable liabilities. This framework fundamentally reframes addiction from a disorder of moral weakness or reward system dysfunction to a developmental trajectory shaped by stress-immune interactions during critical maturational windows (see Fig. 2). The clinical implications are profound: intervention approaches must address inflammatory substrates while targeting behavioral and environmental factors that maintain chronic activation. The therapeutic window during mid-to-late adolescence represents a critical opportunity for prevention and early intervention before vulnerability consolidates into treatment-resistant chronicity. Targeted neuroimmune-modulatory strategies aimed at normalizing maladaptive neuroimmune signaling, when integrated with behavioral and ecological interventions, may improve outcomes relative to approaches focused on reward circuitry alone. Future research priorities include development of inflammatory biomarkers for risk stratification, optimization of combination treatment protocols, and investigation of precision medicine applications based on individual biological profiles. The neuroimmune framework provides a unifying conceptual foundation for these advances while offering hope for more effective and compassionate treatment approaches. Understanding addiction vulnerability as an outcome of developmental adaptation under adverse conditions rather than inherent pathology transform clinical practice from blame to stratified precision, from late rescue to early buffering, and from one-size-fits-all approaches to personalized intervention strategies tailored to individual risk profiles and biological signatures.

## CRediT authorship contribution statement

**Léa Aeschlimann:** Writing – review & editing, Writing – original draft. **Narimane Bouzourène:** Writing – review & editing. **Clara Rossetti:** Writing – review & editing. **Benjamin Boutrel:** Writing – review & editing, Validation, Supervision, Project administration, Funding acquisition, Conceptualization.

## Declaration of competing interest

The authors declare that they have no known competing financial interests or personal relationships that could have appeared to influence the work reported in this paper.

## Data Availability

No data was used for the research described in the article.
